# Applications of Artificial Intelligence in Thrombocytopenia

**DOI:** 10.3390/diagnostics13061060

**Published:** 2023-03-10

**Authors:** Amgad M. Elshoeibi, Khaled Ferih, Ahmed Adel Elsabagh, Basel Elsayed, Mohamed Elhadary, Mahmoud Marashi, Yasser Wali, Mona Al-Rasheed, Murtadha Al-Khabori, Hani Osman, Mohamed Yassin

**Affiliations:** 1 College of Medicine, QU Health, Qatar University, Doha 2713, Qatar; 2 Dubai Academic Health Corporation & Mediclinic Hospital, Dubai 3050, United Arab Emirates; 3 Department of Child Health, Sultan Qaboos University, Muscat 3050, Oman; 4 Hematology Department, AL Adan Hospital, Kuwait City 3050, Kuwait; 5 Hematology Department, Sultan Qaboos University, Muscat 3050, Oman; 6 Hematology/Oncology Department, Tawam Hospital, Abu Dhabi 3050, United Arab Emirates; 7 Hematology Section, Medical Oncology, National Center for Cancer Care and Research (NCCCR), Hamad Medical Corporation (HMC), Doha 3050, Qatar

**Keywords:** artificial intelligence, thrombocytopenia, diagnosis, prognosis, prediction, transmission

## Abstract

Thrombocytopenia is a medical condition where blood platelet count drops very low. This drop in platelet count can be attributed to many causes including medication, sepsis, viral infections, and autoimmunity. Clinically, the presence of thrombocytopenia might be very dangerous and is associated with poor outcomes of patients due to excessive bleeding if not addressed quickly enough. Hence, early detection and evaluation of thrombocytopenia is essential for rapid and appropriate intervention for these patients. Since artificial intelligence is able to combine and evaluate many linear and nonlinear variables simultaneously, it has shown great potential in its application in the early diagnosis, assessing the prognosis and predicting the distribution of patients with thrombocytopenia. In this review, we conducted a search across four databases and identified a total of 13 original articles that looked at the use of many machine learning algorithms in the diagnosis, prognosis, and distribution of various types of thrombocytopenia. We summarized the methods and findings of each article in this review. The included studies showed that artificial intelligence can potentially enhance the clinical approaches used in the diagnosis, prognosis, and treatment of thrombocytopenia.

## 1. Introduction

Thrombocytopenia is a medical condition characterized by low platelet counts. There are many causes thrombocytopenia which can be broadly classified into decreased production, increased sequestration, and increased platelet destruction [1]. The pathogenesis of thrombocytopenia is very complex and can be attributed to a multitude of causes. The decreased production of platelets can be related to bone marrow suppression commonly seen in conditions such as leukemia, patients taking chemotherapy, and in sepsis. Increased sequestration of platelets in the spleen is another major category of conditions that cause thrombocytopenia. Typically, this is seen in patients with splenomegaly, hypersplenism and in patients with portal hypertension. The increase in platelet destruction or utilization in peripheral blood is the final category in thrombocytopenia. Typically, this occurs in conditions where platelets are quickly used up or destroyed by autoantibodies [2]. It is important to note that conditions that cause thrombocytopenia do not exclusively affect one pathological pathway and may involve multiple mechanisms simultaneously such as increased decreased production and sequestration. The diagnosis and treatment of thrombocytopenia require a thorough understanding of the underlying causes and the patient’s medical history. Thrombocytopenia is usually termed based on its causes. For example, when thrombocytopenia is caused by drugs it is termed drug induced immune thrombocytopenia (DITP). Similarly, when caused by sepsis it is termed sepsis associated thrombocytopenia (SAT) [3].

Artificial intelligence (AI) is the simulation of human intelligence by computer programs. These programs are based on a set of algorithms that allow machines to mimic human intelligence through learning and problem solving. Although AI has previously been used in healthcare to automate hospital systems, recently, it has also been utilized in the diagnosis, early detection, and monitoring of diseases [4,5]. In recent years, there have been several successful applications of AI in various medical conditions, such as the diagnosis of LA fibrillation and evaluation of prognosis in COVID-19 [6,7]. This has been made possible because of machine learning (ML), which is a type of AI that utilizes datasets to learn and recognize patterns and create predictions based on them [8]. What makes these algorithms unique is the fact that they can analyze linear and nonlinear variables simultaneously. This allows them to recognize patterns in a very complex manner that can be used to make extremely accurate predictions [4,9]. With the advent of artificial intelligence (AI), healthcare providers can leverage the power of machine learning algorithms and predictive analytics to improve the accuracy and efficiency of thrombocytopenia diagnosis and treatment.

In this review, we aim to summarize the most recent applications related to the use of AI in the diagnosis, prognosis, and mapping for various causes of thrombocytopenia. This review also aims to summarize their methods, performance metrics, limitations and future of each model used.

## 2. Materials and Methods

Our search strategy was developed using PubMed’s Medical Subject Headings (MeSH) terms, along with other title and abstract keywords. For our disease of interest (thrombocytopenia) we included terms related to thrombocytopenia and its subtypes such as “Immune Thrombocytopenia”, “Immune Thrombocytopenic Purpura”, “ITP”, “thrombocytopenia”, “Drug induced thrombocytopenia”, “Viral thrombocytopenia”, and other terms of this nature to avoid missing any related articles. To include articles discussing the used of AI in thrombocytopenia we also included terms for machine learning (ML) such as “artificial intelligence”, “machine-learning”, and “AI”. This research was not restricted by a language or timeframe. A polyglot translator was used to convert the initial search strategy to Embase, Web of Science, and Scopus [10]. All the studies identified by the search strategy were moved into EndNote, where duplicates were removed. The remaining studies were then moved into Rayyan to remove any remaining duplicates and start the screening process.

This review included original research articles that discuss the use of ML algorithms in the various types of thrombocytopenia in humans. Full-text articles submitted abstracts and conference abstracts were all included within our study. Studies were excluded from our study for the following reasons: (1) animal studies, (2) reviews or non-original articles, and (3) non-English articles.

The data that has been collected in this paper includes the type of study; publication year; outcome assessed; method used to create models; model utilized; model evaluation metrics (sensitivity (SEN), specificity (SPE), accuracy (ACC), and area under the receiver operating curve (AUC)); strengths; and limitations. The AUC for the models were classified into unsatisfactory (<0.6), satisfactory (0.6 to <0.7), good (0.7 to <0.8), very good (0.8 to <0.9), and excellent (0.9 to 1.0). If more than one model was utilized in the study, the metrics for the best preforming model(s) were extracted.

## 3. Results

**Figure 1:**
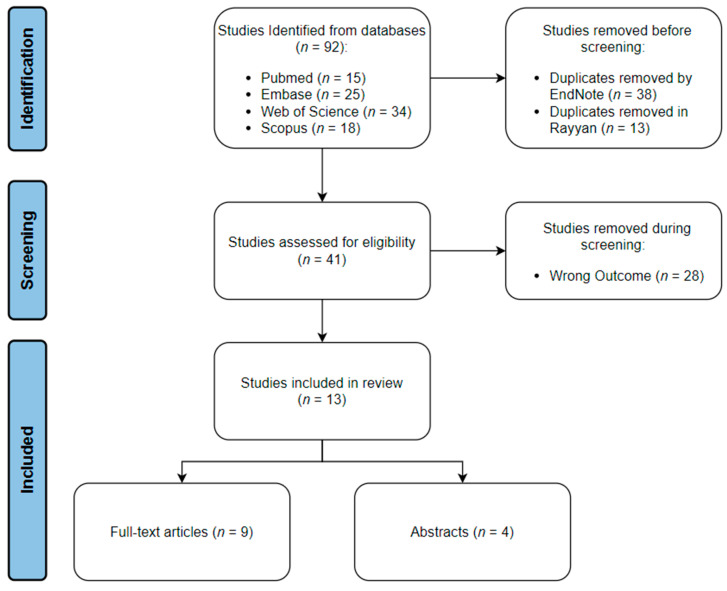
Schematic representation of review process.

The search strategy yielded a total of 92 articles across all four databases. All the studies were imported into EndNote, where 38 duplicates were automatically identified and deleted. The articles were then transferred to Rayyan, where 13 more duplicates were manually identified and removed. The inclusion–exclusion process was done using Rayyan. Twenty-five articles were excluded from the study due to not meeting our inclusion criteria. Sixteen articles were included in the review; of which, nine were full-text articles, and seven were abstracts only. The schematic representation for the processes of identification, screening, and inclusion is shown in (Figure 1).

The studies included within our paper covered five types of thrombocytopenia (Sepsis-Associated Thrombocytopenia (SAT), Drug-Induced Immune Thrombocytopenia (DITP), Severe Fever with Thrombocytopenia Syndrome (SFTS), Immune thrombocytopenia (ITP), and unspecified Hospital-Acquired Thrombocytopenia (HAT)). Under each topic, studies were categorized into diagnostic, prognostic, and predictive where applicable. Data collected on the outcomes, advantages and disadvantages of the ML models, can be seen in Table 1. Performance matrices for the best-preforming ML model(s) in each study can also be seen in Table 2.

### 3.1. Sepsis Associated Thrombocytopenia

Sepsis is defined as a life-threatening condition characterized by organ dysfunction due to a dysregulated immune response to an infectious agent [20]. It is referred to as “septic shock” when circulatory and cellular metabolic abnormalities become present leading to a considerable increase in morbimortality [21]. Almost 50% of patients with sepsis in the intensive care unit (ICU) develop thrombocytopenia, termed as SAT [22,23]. The mechanisms behind SAT are believed to be complex but are mainly associated with bone marrow suppression accompanied by endothelial dysfunction. This combination results in reduced production of new platelets and increased utilization of platelets due to disseminated intravascular coagulation and systemic inflammation. Clinically, the development of thrombocytopenia in sepsis patients is an indication of poor prognosis. Hence, the use of AI for early identification and risk prediction in these patients can be of great value. The utilization of ML in the prediction of poor outcomes in critically ill patients in the ICU has become very common in the literature and is showing a lot of promise in ensuring better patient care [24,25]. Unfortunately, the utilization of thrombocytopenia in these studies is very limited, although it has been shown to be a very important predictor of poor prognosis in ICU patients with sepsis and is part of the Sepsis-related Organ Failure Assessment (SOFA) [21,23,26]. Nevertheless, studies that have looked at SAT in critically ill patients have identified possible application for ML in them.

#### 3.1.1. Diagnosis

The early detection of SAT is extremely important in a clinical setting as research has shown that platelet transfusions protect these patients from possibly fatal bleeding [27]. However, the issue with conventional monitoring of platelet count is that by the time SAT is identified in the patient the patient remains at risk of severe bleeding for a few days before receiving platelet transfusions due to the delay between diagnosis and transfusion [21]. Hence, ML algorithms that track platelet count changes in patients can provide a major advantage for them, as it allows for early detection of patients at high risk for SAT.

A recent publication by Jiang X and others attempted to address this issue by utilizing four ML algorithms (Random Forest (RF), Bayes (Bayesian), Neural Network (NNET), and Gradient Boosting Machine (GB)) to assess the decrease in platelet count, as well as other variables in ICU patients suffering from sepsis for the early detection of patients at risk of thrombocytopenia or severe thrombocytopenia. A total of 1455 ICU sepsis patients were included in the study, of which 49.7% developed thrombocytopenia. The sample population was divided into training and testing sets in a 7:3 ratio, and each ML algorithm was cross-validated 10 times [11]. Backward selection was utilized to select the 10 most important predictors out of 57 variables for the prediction of thrombocytopenia and severe thrombocytopenia. Each ML algorithm was externally validated by applying it on an online dataset from Medical Information Mart for Intensive Care III (MIMIC III) [11,28].

**Figure 2:**
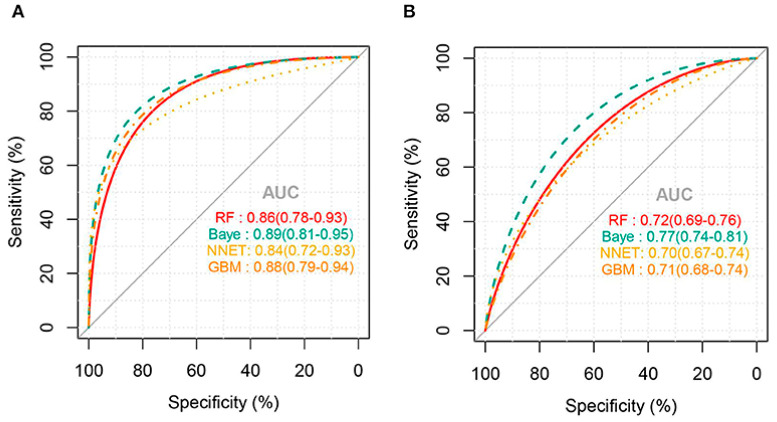
AUC for ML models predicting severe thrombocytopenia: (**A**) internal validation and (**B**) external validation (Jiang, X. et al., 2022) [11].

External validation of the models for predicting thrombocytopenia showed that the NNET and GB models had the best predictions with a good AUC of 73% and 72%, respectively. There were no statistically significant differences between the two models at predicting thrombocytopenia in sepsis patients. Meanwhile, the RF and Bayes models had poorer predictive ability with an area under the ROC of 63% and 54%, respectively. Confusion matrix results for thrombocytopenia prediction showed that NNET had the highest precision and accuracy of 0.68 and 0.71, respectively. For the prediction of severe thrombocytopenia, the AUC for all ML algorithms was higher than that of thrombocytopenia prediction (Figure 2). External validation of the models for predicting severe thrombocytopenia showed that the Bayes model had the best predictive ability with a good AUC of 77% [11].

The results of this paper suggest that ML algorithms can prove to be beneficial in prediction of SAT and severe thrombocytopenia and hence would assist in the early management of the patients at risk. However, it is important to mention the fact that it was a single-center study. The utilization of multiple centers for the ML algorithm could have provided greater predictive ability for the model and would make the model more applicable externally. Missing data was an issue in the paper that was addressed using multiple imputation which assumes that data is missing at random which might not be the case. Adding to that, the exact cause of thrombocytopenia in the sepsis patients was not verified, as some patients developed thrombocytopenia due to drug treatment and not sepsis yet, were still included within the study. As a result, the models produced are not exclusively predicting thrombocytopenia/severe thrombocytopenia caused by sepsis, and their results could be affected by other causes of thrombocytopenia. Since the models used were non-interpretable, the models cannot explain the associations between the variables identified and the prediction of thrombocytopenia/severe thrombocytopenia.

#### 3.1.2. Prognosis

As stated previously, thrombocytopenia is associated with poor prognosis in sepsis patients. Hence, it is important to properly assess the prognosis of patients with SAT to ensure proper care for these patients. Recent studies have shown that red cell distribution width (RDW) is a possible indicator for poor prognosis in various cancers and cardiovascular disease [29,30,31]. Several studies have also shown that RDW has significant clinical utility as an independent predictor of poor prognosis in critically ill patients with sepsis and SAT. RDW is reliable in reflecting the levels of systemic inflammation in these patients [32,33,34]. Since RDW is routinely measured clinically in a relatively inexpensive process, it could prove to be a useful ML marker for assessing prognosis in sepsis patients with SAT.

A recent publication by Ling J et al. has utilized eXtreme Gradient Boosting (XGBoost), a ML algorithm, to predict the 28-day mortality risk for sepsis patients based on 15 variables including RDW. A total of 809 patients with sepsis were retrospectively selected from the MIMIC-III database and were divided into thrombocytopenia group (471 participants) and control group (338 participants) based on their platelet count. Eight laboratory variables, four disease-related variables, and two demographic variables were used to create a prediction model for their 28-day survival. Thrombocytopenia group was further subdivided into survivors and non-survivors. Using a 3:1 ratio, the dataset was randomly divided into training and testing sets. SHapely Additive exPlanations tool (SHAP) was used to interpret the prediction model and reliably compute the contributions made by each variable to the model, as well as rank them based on importance [12].

The results of the paper showed that 28-day mortality in sepsis patients with thrombocytopenia was significantly higher than those without thrombocytopenia at the baseline (48.2% vs. 38.5%, respectively). SHAP interpretation of the XGBoost indicated that RDW was the second most important predictor of 28-day mortality in these patients following the SOFA scores. When comparing the subgroups of thrombocytopenia through AUC analysis, RDW was shown to be the most important predictor of 28-day mortality in thrombocytopenic patients with an area under the ROC of 0.646. An RDW of 16.05 displayed the best sensitivity and specificity for the prediction of mortality in these patients (70% and 57%, respectively) [12].

One of the limitations for the model was the fact that there was missing data that could have possibility limited the model prediction accuracy. Adding to that, the model was created utilizing retrospective single-center data, which is not ideal. The AUC of the model was only 0.646 and does not provide enough discriminatory ability at predicting the 28-day mortality clinically. However, a relatively low AUC is expected due to the fact that a very small number of predictor variables were used. Hence, the model proposed by Ling and others can prove to be more useful if more variables were included in the model that would provide a higher predictive ability for the 28-day mortality.

### 3.2. Drug-Induced Immune Thrombocytopenia

DITP is a common life-threatening complication seen in patients taking multiple drugs at the same time [35]. This clinical syndrome is typically associated with severe bleeding that could ultimately result in death. There are many pathological mechanisms discussed in the literature to explain the causative mechanisms behind DITP [35,36,37,38]. However, the most widely accepted mechanisms are direct bone marrow suppression by drugs and the development of drug-dependent antibodies (DDAbs) that activate platelets, ultimately leading to their depletion [38]. The main dilemma with DITP is that it is challenging to diagnose clinically, and it is even more problematic to identify the causative drug [39]. Moreover, the current experimental invitro methods of DITP diagnosis through DDAbs are unreliable, expensive, time-consuming, and are only available in a few platelet-specialized laboratories [40]. Currently, the most effective approach for the treatment of patients with DITP is the cessation of the causative drug [41]. Hence, the early diagnosis and detection of the causative drug in patients with DITP is extremely important. ML that utilizes in silico methods has shown to be a possible cost-effective and timely approach for the diagnosis of DITP.

#### 3.2.1. Diagnosis of DITP

Linezolid is a synthetic antimicrobial used in the treatment of infections. Several studies have shown that linezolid could lead to linezolid associated thrombocytopenia (LAT) a type of DITP [42,43,44]. The identification of LAT in patients taking linezolid treatment for infections may be difficult and time-consuming. To address this, Takahashi and colleagues conducted a study to create a classification tree model that predicts LAT in patients taking linezolid treatment. A total of 74 patients receiving linezolid treatment were retrospectively included in the study. LAT was defined as a 25% decline in platelet count from baseline. The baseline platelet count; age; total linezolid concentration; platelet count changes at 24, 48, 72, 96, and 120 h; creatinine clearance; and body weight were used as predictors for LAT in these patients. Binary decision trees were used to utilizing different combinations of the predictor variables to create tree models for LAT prediction. These trees are ML algorithms that classify observations by creating a sequence of binary questions. Binary questions are formed by creating the best possible splits for the data and repeats till further branching no longer improves the classification of observations. Trees were then pruned to avoid overfitting of the model to the learning data. The tree model with the lowest misclassification rate was taken as the final model [13].

**Figure 3:**
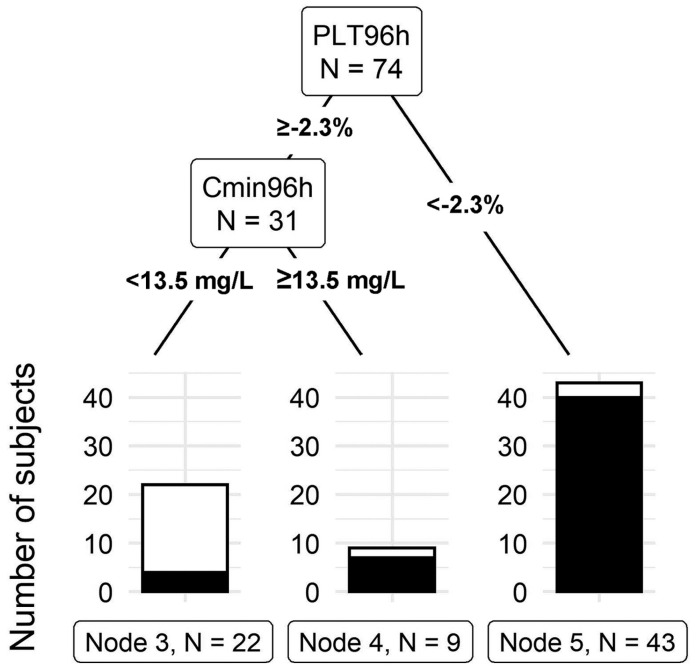
Classification tree for thrombocytopenia prediction. PLT96h: Platelet change from the baseline at 96 h after the initial dose. Cmin96 h: linezolid total concentration at 96 h after the initial dose (Takahashi, S. et al., 2021) [13].

The model that included the total linezolid concentration, platelet count at 96 h, total body weight, age, gender, baseline platelet count, and creatinine clearance outperformed all other tree models with a misclassification rate of 12.2%. This final tree model can be seen in (Figure 3). The first split in the model is at 2.3% platelet count from the baseline at 96 h. Forty-three individuals had a 96-h platelet count less than 2.3% of the baseline, of which 40 developed thrombocytopenia by day 14. The second split occurred at a linezolid total concentration cut-off of 13.5 mg/L at 96 h. Nine out of the thirty-one subjects had a linezolid concentration above 13.5, of which seven developed thrombocytopenia by day 14. These findings indicate that, at 96 h, a drop in platelet count to less than 2.3% or having a linezolid total concentration above 13.5 indicates a high risk of LAT by day 14. The final model had a sensitivity of 92.2% and a specificity of 78.3%. Leave-one-out validation of the final model indicates a cross-validation error of 20.3% [13].

The model presented by Takahashi and colleagues is unique in the fact that it’s the only study in our paper that utilized CART in the identification of predictors for LAT. The model generated clinically relevant cutoffs for the prediction of LAT. Moreover, they have utilized variables in their prediction that can be easily obtained from patients starting on linezolid treatment which makes it easy and simple to apply clinically. However, renal function is a very important predictor for LAT but was not included in the prediction model. The model generated was also not externally validated which is very important to understand the generalizability of such a model. Adding to that, the model utilized retrospective data which is limited in terms of predictors and tends to have more missing data.

Maray and colleagues took a different approach in creating a predictor model for LAT. Data on a total of 46,520 patients admitted to the ICU were extracted from the MIMIC-III database [28]. Thrombocytopenia was defined as a 50% decrease in platelet count from baseline and this cut-off was used to divide the patient population into with and without thrombocytopenia. The dataset was divided into training and testing sets in an 8:2 ratio. Univariate analysis was utilized to compare demographic variables, duration of linezolid treatment, SOFA scores, and many other variables between both groups. Variables that were found to be significantly different in both groups were included in the first predictor model for multivariable logistic regression. A second model was also created using backwards elimination to select the most important predictors for this model. In the training set, both models had similar performances with the first model having an AUC of 0.89 compared to 0.88 in the second. The accuracy, sensitivity, and specificity were the same between both models (0.79, 0.80, and 0.71, respectively). On the testing set however, the second model slightly outperformed the first with an AUC of 0.80 compared to 0.77. It also had a higher accuracy (0.75 vs. 0.73), sensitivity (0.78 vs. 0.76), and specificity (0.62 vs. 0.60) [14].

The models presented by Maray and colleagues both showed good metrics at predicting LAT and, similarly to Takahashi, utilized variables that are easy to obtain in patients who are just started on linezolid therapy. However, Marray and colleagues did include renal function in their predictor model. They also included hepatic function in their model. Both renal and hepatic functions are important in predicting LAT since linezolid metabolism is dependent on these organs. The early detection of LAT allowed for physicians to maintain patient safety by switching antibiotics when the platelet count drops to a critical level. This can be made possible through ML models presented by Marray or Takahashi.

Another drug that is commonly associated with DITP is heparin. Approximately 1–3% of all patients treated with heparin develop HIT, and similarly to all other types of induced thrombocytopenia, it is a life-threatening condition [45]. A major issue seen in the normal diagnostic approach for DITPs is that misdiagnosis is common due to lack of diagnostic data utilized [46,47]. A study by Nilius and others utilized ML algorithms that integrate clinical and laboratory information to diagnose HIT more accurately than the traditional approach by the American Society of Hematology (ASH) [15]. A total of 1393 patients with suspected HIT were included in their study. As a reference, standard platelet washed heparin induced platelet activation (gold standard for diagnosing HIT) was used. Based on literature review, they selected several possible predictor variables for HIT. These included clinical variables (degree of thrombocytopenia, timing of thrombocytopenia, etc.), as well as laboratory variables (immunoassay detecting anti-PF4/heparin antibodies, hemoglobin concentration, WBC count, platelet count, etc.). Three immunoassay tests were conducted on serum samples collected from the patients (IgG-specific ELISA, particle–gel immunoassay (PaGIA), and chemiluminescent immunoassay (CLIA)). Patients were randomized by HIPA results then divided into a training and validation set in a 75:25 ratio. While creating the model, the researchers accounted for the practical application of the model by ensuring that all variables used were readily available, timely, consistent, and easy to collect. Backwards stepwise selection was used to select the most important variables in the model [15].

**Figure 4:**
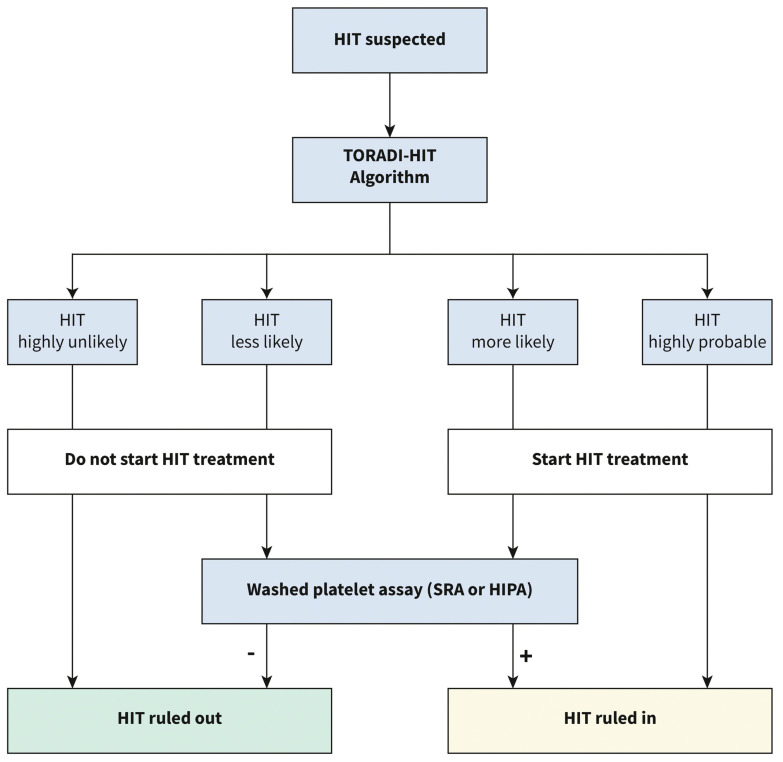
Proposed diagnostic algorithm for heparin induced thrombocytopenia: TORADI−HITP (Nilius, H. et al., 2022) [15].

Five different ML models were trained for each immunoassay (logarithmic regression, elastic net logarithmic regression, gradient boosting machine, random forest, and support vector machine). The support vector machine model outperformed all others in CLIA (AUC of 0.989). The gradient boosting machine model outperformed all others in PaGIA (AUC of 0.991). The support vector machine model outperformed all others in ELISA (AUC of 0.985). When the models were applied to the validation set the sensitivity of the models were 96%, 100%, and 89% for the CLIA, PaGIA, and the ELISA models, respectively. The specificity was 95% for all models. These performance matrices outperformed the current metrics for the recommended algorithm for the diagnosis of HIT, and hence, a new algorithm was suggested by the authors (Figure 4) [15].

#### 3.2.2. Predicting Drugs Causing DITP

Wang, B. et al. developed a several models to predict whether a drug could lead to DITP using seven different ML methods [16]. A DITP dataset was collected from an online database, “platelets on the web”, which contained information on the compounds tested with DDAb [48]. Compounds that had detectable DDAbs were classified as DITP toxicants (93) and those without DDAbs as non-toxicants (132). The dataset was then randomly divided in an 8:2 ratio into training and external validation sets, respectively. Support vector machine (SVM), k-nearest neighbor (k-NN), RF, naive bayes (NB), artificial neural network (ANN), adaptive boosting (AdaBoost), and XGBoost were used to produce binary classification models for DITP. Hyperparameters for each model was optimized by five-fold cross-validation, and the variance was reduced by 10× cross-validation. These models utilized six molecular fingerprints and three molecular descriptors to predict whether or not a drug can cause DITP [16].

Over 828 models were created using different combinations of molecular descriptors and fingerprints in the 7 ML algorithms. k-NN and XGBoost outperformed all other ML models in predicting DITP toxicants. The k-NN model combining molecular features of RDMD + PubChem netted the highest prediction performance with an AUC of 0.628, accuracy of 62.7%, sensitivity of 69%, and specificity of 56.6%. In simpler terms, this model was the best at distinguishing DITP toxicants from DITP non-toxicants based on their molecular fingerprints and descriptors. On external validation, the k-NN RDMD-PubChem model still outperformed all other models with an AUC of 0.769, accuracy of 75.6%, sensitivity of 83.3%, and specificity of 70.4%. This shows that this model is good at distinguishing DITP toxicants from non-toxicants [16].

Based on this information, the k-NN model using RDMD-PubChem can be used as an alternative tool to the qualitative methods used to predict DITP toxicity. However, it is important to mention that this model utilized a small sized dataset with a limited number of descriptors. Hence, it is unable to classify all agents accurately especially stereoisomers, tautomeric forms, and protonation states molecular features on these were absent in the original dataset. Other than that, the model displays sufficient performance matrices on external validity to distinguish DITP toxicants from non-toxicants with good discriminatory ability.

### 3.3. Hospital Acquired Thrombocytopenia

A common bleeding disorder following surgery is hospital acquired thrombocytopenia (HAT), This form of thrombocytopenia can be attributed to some of the previously discussed topics such as DIT or SAT. In a research paper conducted by Cheng and others, 7 ML models (GB, RF, logistic regression (LogR), XGBoost, multilayer perceptron, SVM, and k-NN) were created to predict patients at risk of HAT following surgery. Adult patients who were administered to the ICU following surgery were included in the study and divided in a 7:3 ratio into training and testing sets, respectively. Ten-fold validation was performed on the models generated. From all the included patients, 13.1% developed thrombocytopenia. The results of internal validation showed that the RF and GB models outperformed all other models in the prediction with an AUC of 0.834 and 0.828, respectively, and there were no statistically significant differences between the two models. Both models had a high sensitivity of 79.3% and 73.6%, respectively. Specificity for the models were 79.1% and 73.7%, respectively [17].

The models presented by Cheng and colleagues show very good performance matrices for the prediction of HAT. Unfortunately, these models were not externally validated and so their generalizability to other patient populations cannot be evaluated. The models were also generated utilizing single-center retrospective data which limits its generalizability and suffers from missing data and insufficient variables.

### 3.4. Immune Thrombocytopenia

Immune thrombocytopenia (ITP) is an autoimmune bleeding disorder [49]. It is clinically characterized by low platelet count and the presence of autoantibodies to platelets [50]. Diagnosis is typically made through the exclusion of all other causes of thrombocytopenia. ITP is considered a self-limiting disease in children where prognosis is good, and remission occurs easily. In adults however, ITP is chronic with a higher mortality rate [51,52]. The pathogenesis of ITP is not well understood but involves the formation of IgG autoantibodies that target the glycoproteins IIb-IIIa on platelets. This results in the phagocytosis of these platelets hence leading to a drop in circulating platelet count and, ultimately, thrombocytopenia [50]. Thrombopoietin receptor agonists and corticosteroids are most commonly used in the treatment of ITP [53].

#### 3.4.1. Diagnosis

Kim, T. and others used a clinical database to create five ML models (RF, NB, LogR, SVM, and AdaBoost) to predict chronic immune thrombocytopenia in pediatric patients with ITP. A total of 969 pediatric patients with ITP were included in the study, of which, 332 had confirmed acute ITP and 253 with chronic ITP. Clinical (age, gender, race, ethnicity, presence of primary ITP) and laboratory variables (baseline platelet count, leukocyte count, lymphocyte count, eosinophil count, mean platelet volume, anti-nuclear antibody, immature platelet fraction, direct antiglobulin test, and immunoglobin levels) were used in the ML models to predict chronic ITP and 10-fold cross-validation was performed. The 100-tree random forest model outperformed all other models in predicting chronic ITP (AUC: 0.795, accuracy: 0.737, precision: 0.738, F1-score: 0.671, and recall: 0.737). Naïve Bayes was the second-best preforming model (AUC: 0.792, accuracy, 0.698, precision: 0.737, F1-score: 0.671, and recall: 0.698) [54].

This study shows that clinical and laboratory information at the time of ITP diagnosis can be used to predict the development of chronic ITP in these patients. This is important because almost 1 of every 4 kids with ITP develop chronic ITP. Hence, models that can predict the development of chronic ITP early on would result in earlier treatment in these patients and ultimately, better health outcomes. However, it is important to note that then models presented by Kim and colleagues were not externally validated. Consequently, we cannot assess the generalizability of this model on other pediatric populations with ITP. Similar to the other studies mentioned previously, the models were generated using retrospective data, which has some limitations discussed earlier.

#### 3.4.2. Prognosis

One of the main indicators for poor prognosis in patients with any form of thrombocytopenia is bleeding. A study by An, Z. Y. and others developed ML models to predict the risk of critical bleeds in patients with ITP. Data from eight centers in China were used to create this model utilizing nine predictor variables (Platelet count, age, onset of ITP, infection, type of ITP, bleeding of skin and mucosa, low platelet count, cardiovascular disease, and uncontrolled diabetes). Out of all the models, the RF model outperformed all others (AUC: 0.901 when externally validated) [55]. The models were then further verified in a prospective cohort of 37 centers in China. The RF model had an AUC of 0.776, which demonstrates good predictive ability for the risk of critical bleeds.

This study is the only study included in our paper that has been verified clinically in a prospective cohort. An and colleagues utilized multi-center data to create their model and externally validate them, which makes it stand out from most other papers as they utilize single-center data, which is limited. Prospective cohort verification of the models allows for the evaluation of ML models in a real-world setting, which is essential for determining their effectiveness in practical applications. It also insures that the models generated were not just overfitted for the data that was used to generate them.

Another study by Zhang, X.H and others created 10 ML algorithms (SVM, k-NN, LogR, linear discriminant analysis, decision tree, RF, GB decision tree, AdaBoost, XGBoost, and light gradient boosting machine) to predict the 30-day mortality in patients with ITP with intracranial hemorrhage. A 10-fold cross-validation was performed in the training cohort then external validation was performed in 11 different centers. The SVM model outperformed all other models for the prediction of 30-day mortality on internal validation (AUC: 0.879, F-1 score: 0.748, and sensitivity: 0.600) [56].

The model proposed by Zhang and colleagues utilized 16 centers in the creation and internal validation of their models. Training machine learning (ML) models on multiple centers, also known as multi-center training, is important, because it improves the generalizability of these models because it provides a more diverse range of data, which can help the model learn patterns and relationships that are applicable across different settings. They also externally validated the models on 11 independent centers, which ensures that the models they have created can be generalized to other settings. Unfortunately, the research did not report the AUC for the externally validated models in their conference abstract.

A final study by Liu, F. Q. and others looked at the use of RF classifier in the prediction of relapse in patients with IPT after the cessation of corticosteroid treatment. Seventy-fie fecal samples (15 after corticosteroid cessation and 60 before) from 60 patients with ITP were obtained and analyzed. The pre-corticosteroid group was classified into responsive, and relapse based on their response to corticosteroids 3 months later. Using shotgun meta-genomic sequencing, the microbial biomarkers from thirty samples (training set) for relapse/resistance were identified and included in the ML prediction model. The model was validated on thirty other samples. AUC for the random forest classifier model was 0.87 [57]. These models were not externally validated, so it is not possible to assess their generalizability.

### 3.5. Severe Fever with Thrombocytopenia

Severe fever with thrombocytopenia syndrome (SFTS) is an infectious zoonosis currently emerging in the Southeast Asia region. It is caused by the Phlebovirus from the Bunyaviridae family but is more commonly known as the severe fever with thrombocytopenia syndrome virus (SFTSV). It is believed to be transmitted through ticks to humans, however some human-human transmissions have been recorded through body fluids [58,59,60,61]. Clinically, patients with SFTS typically present with high fever, thrombocytopenia sometimes accompanied by leukocytopenia. In more severe cases, however, sever hemorrhagic fever, encephalitis and multiple organ failure can occur. The latter symptoms are associated with increased risk of death in these patients [60]. Due to the nature and severity of this syndrome, it is important to limit the transmission of this disease. Hence, recent studies have utilized various ML methods to predict the transmission of SFTS to better control the disease [62].

Miao and others worked on a ML algorithm to predict the potential distribution for SFTS in a two-step process. The first step utilized modelling techniques to map out the distribution of H longicornis, a tick that is involved in the transmission of SFTSV. This was done using data on known locations for this species of ticks. An ecological niche (EN) modelling technique was used for the mapping of the distribution of H longicornis based on 51 ecogeographical and climatic variables. Based on the EN model, 10 variables were identified to be important predictors and the predicted global distribution of H longicornis was created. In the second step, potential transmission hotspots for SFTS were predicted using a boosted regression tree (BRT) model that utilized the previous model predictions. BRT was suitable for this since it is able to predict organism distribution while accounting for nonlinearity and incorporating risk factors into the prediction. The AUC for the BRT model prediction performance is satisfactory with AUCs of 0.976 for the training datasets and 0.893 for the testing datasets [18].

**Figure 5:**
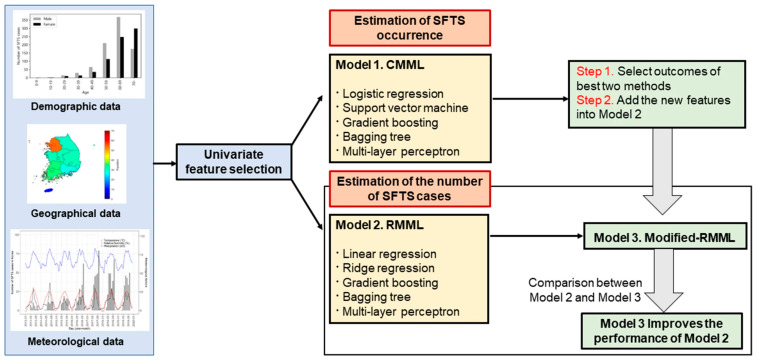
Schematic diagram for model predictions (Cho, G., S. Lee, and H. Lee 2021) [19].

An article by Cho and others employed ML algorithms to predict the transmission of SFTS in 7 different geographical locations in Korea. They utilized ML algorithms in 3 different models: Classification model in machine learning (CMML) to predict the occurrence of SFTS, Regression model in machine learning (RMML) to predict the number of SFTS cases, and a modified RMML model that incorporated the results from CMML into RMML to more accurately predict the number of SFTS cases. For the CMML model 5 ML algorithms were utilized (LogR, SVM, GB, Bagging Tree, and Multi-layer Perception). For the RMML model, 5 ML algorithms were used (Linear regression, Ridge regression, GB, Bagging Tree, and Multi-layer perception). Geographic, demographic, and meteorological data were utilized to create the models. Data from 2016 to 2018 were used to train the models and data from 2019 were used to evaluate the models. A univariate analysis was used to select the most important predictors for each model. Features from the best two algorithms in the CMML model (BGR and BTR) were included in the RMML model to create the modified RMML model. The schematic used in model creation is shown in Figure 5. The modified RMML model improved the predictions for the number of SFTS cases and reduced the mean squared error in the training set from 79.2% to 40.6% and in the testing set from 52.2% to 12.6%. The BTR ML model from the modified RMML was superior to all others. The AUC for the BTR model was >0.9 at predicting the occurrence of SFTS [19].

## 4. Discussion

The aim of this review was to investigate the different applications of ML in thrombocytopenia that are currently available. We have found a limited number of studies that have utilized AI mainly in the diagnosis and prognosis of thrombocytopenia. We have shown that AI can also be utilized in the detection of drugs that can cause DITP, and mapping of potential hotspots for SFTS transmission. A very large number of the included studies showed high predictive ability with an AUC >0.8. The available evidence in this review paper suggests that AI may be effective in predicting and evaluating the prognosis of patients with thrombocytopenia.

It is still important to note that the research into the applications of AI in thrombocytopenia is very limited in number. However, when looking at other conditions where AI is more commonly used, it can be seen that AI is effective in the diagnosis prognosis and even treatment of patients. In leukemia for example, multiple papers have shown that AI can accurately diagnose and subtype leukemia using image processing technologies [63,64]. Other studies have even utilized AI in the histopathological identification, subtyping, and metastasis of breast cancers [65,66,67].

Frequently, papers included in this study utilized retrospective data in the generation of their ML models. Although these data are convenient and easy to acquire, they also have several disadvantages that can affect the accuracy and usefulness of these models. For example, the data may be collected in a way that is not consistent or standardized, leading to inaccuracies in the model. They also typically suffer from incomplete data, which was seen repeatedly in this study. Some models are unable to include all the important predictors due to the fact that the original dataset does not have data on these variables. Hence, we hope to see more future studies that utilize prospective data rather than retrospective data to address this issue, as this addresses the previously mentioned issues.

Another issue that was commonly noted in the articles included in this review was the fact that some models had very high-performance matrices on internal validation. Although this may seem appealing, but it may have been due to the overfitting of these models to their datasets. When this issue is combined with the fact that many of the studies utilized single-center data, these models become less generalizable. Hence, there is a great need for future studies using ML to utilize more robust databases with large sample sizes that are representative of the population in order to avoid overfitting. This issue commonly faces in ML studies in general, not just the ones targeting thrombocytopenia [68,69]. This issue can also be addressed by moving towards more clinical implementation of ML models rather than just testing them on other retrospective datasets. This will ensure that these models are effective clinically and can be used in a clinical setting.

## 5. Conclusions

In conclusion, recent advances in the use of AI in predicting the diagnosis and prognosis of several causes of thrombocytopenia have been summarized in this review. We showed that ML algorithms can possibly provide a faster, reliable evaluation of patients with thrombocytopenia and, in some cases, may even outperform the current approaches for evaluating the diagnosis and prognosis of patients. With further research, AI can possibly revolutionize future medical practice. However, many of the ML models discussed in this study need to be further validated and improved by including more predictors and using prospective data rather than retrospective data in the training and testing of these ML models. Adding to that, more ML models that assess different aspects of patient care such as treatment response require further investigation.

## Figures and Tables

**Table 1:** Data extraction summary for full-text articles included.

Study (Year)	Outcome	Advantages	Disadvantages
Jiang, X., et al. (2022) [11]	Predicting SAT and severe SAT	Internally and Externally Validated Very good AUC for severe SAT internally (0.84–0.88) Good AUC for severe SAT externally (0.74–0.79) Multiple ML models utilized	Single-center retrospective study Missing Data Other causes of thrombocytopenia were not accounted for Models cannot explain the relationships causing thrombocytopenia
Ling, J., et al. (2021) [12]	Predicting the 28-day mortality risk in patients with SAT	Utilized Interpretable ML algorithm Analyzed multiple predictors of poor prognosis	Single-center retrospective study Missing Data Not all confounders were identified Model is not validated externally
Takahashi, S., et al. (2021) [13]	Predicting LAT in patients taking linezolid treatment	High sensitivity (0.922 for final model) Identified clinically important cut-offs for LAT	Retrospective study Model is not validated externally Limited dataset Model did not account for patient’s background and renal function
Marray, L., et al. (2022) [14]	Predicting LAT in ICU patients started on linezolid treatment	Variables used in the models are easy to obtain and cheap Large sample size used to create the models Very good AUC for both models internally (0.89 and 0.88)	Models not externally validated Models do not account for other causes of thrombocytopenia
Nilius, H., et al. (2022) [15]	Diagnosing HIT based on clinical and laboratory data	All models had excellent AUC internally (>0.98) All models had very high sensitivity (>0.89) All models had very high specificity (>0.95) Model outperformed current diagnostic algorithm for HIT diagnosis Large sample size was used to create the models	Majority of patients included were from Switzerland Models not externally validated
Wang, B., et al. (2022) [16]	Distinguishing DITP toxicants from non-toxicants	Internally and Externally Validated Multiple ML models utilized Good AUC (0.769 for externally validated best model) High sensitivity (0.833 for externally validated best model)	Missing Data Model cannot be generalized for every toxicology screen Limited number of variables Limited dataset
Cheng, Y., et al. (2021) [17]	Prediction of HAT risk in patients following surgery	Good AUC for the models (>0.70) Models used easy to access variables	Single-center retrospective study Low number of predictors Models not externally validated
Miao, D., et al. (2020) [18]	Mapping global potential hotspots for SFTS Transmission	Very good AUC for the models (>0.89) Multiple ML models utilized	Inaccuracies in human case report data Some important predictors were not considered (e.g., density of certain types of ticks)
Cho, G., S. Lee, and H. Lee (2021) [19]	Mapping SFTS Virus Transmission in South Korea	Excellent AUC in training set (>0.98) and in testing set (>0.95) Multiple ML models utilized Model utilized many variables in the prediction	Model cannot identify asymptomatic/mild/subclinical cases Not enough laboratory information to form a sufficient system Environmental factors may affect the model Transmission of SFTSV is not well understood and hence could also affect the model

SAT, sepsis associated thrombocytopenia; DITP, drug induced immune thrombocytopenia; LAT, linezolid associated thrombocytopenia; HAT, heparin induced thrombocytopenia; HAT, hospital acquired thrombocytopenia; and SFTS, severe fever with thrombocytopenia syndrome.

**Table 2:** Performance metrics for the best models in the included full-text articles.

Study (Year)	Outcomes	Best Models	Validation	AUC	ACC	SEN	SPE
Jiang, X., et al. (2022) [11]	Predicting Thrombocytopenia	NNET	Internal	0.79	NR	NR	NR
			External	0.72	0.68	NR	0.71
	Predicting Severe Thrombocytopenia	Bayes	Internal	0.89	NR	NR	NR
			External	0.77	0.68	NR	0.62
Ling, J., et al. (2021) [12]	Prognosis of SAT	XGBoost based on RDW	Internal	0.646	NR	0.70	57
Takahashi, S., et al. (2021) [13]	Predicting LAT	CART	Internal	NR	NR	0.922	0.783
Maray, I., et al. (2022) [14]	Predicting LAT	LogR Model 1	Internal	0.89	0.79	0.71	0.80
		LogR Model 2	Internal	0.88	0.79	0.71	0.80
Nilius, H., et al. (2022) [15]	Diagnosing DIT using ELISA	SVM	Internal	0.985	NR	0.89	0.95
	Diagnosing DIT using CLIA	XGBoost	Internal	0.989	NR	0.96	0.95
	Diagnosing DIT using PaGIA	SVM	Internal	0.991	NR	1.00	0.95
Wang, B., et al. (2022) [16]	Predicting DITP toxicity of drugs	k-NN based on RDMD-PubChem	Internal	0.628	0.627	0.69	0.566
			External	0.769	0.756	0.833	0.704
Cheng, Y., et al. (2021) [17]	Predicting HAT following surgery	RF	Internal	0.834	NR	0.793	0.791
		GB	Internal	0.829	NR	0.736	0.737
Miao, D., et al. (2020) [18]	Predicting Potential transmission of SFTS	BRT	Internal	0.893	NR	NR	NR
Cho, G., S. Lee, and H. Lee (2021) [19]	Predicting SFTS Occurrence	GB	Internal	0.986	NR	NR	NR
		BT	Internal	1.00	NR	NR	NR

NNET, neural network; Bayes, Bayesian; XGBoost, extreme gradient boosting; k-NN, k-nearest neighbor; CART, classification and regression tree; LogR, logistic regression; SVM, support vector machine; RF, random forest; GB, gradient boosting; BT, bagging tree; BRT, boosted regression tree; AUC, area under ROC curve; ACC, accuracy; SEN, sensitivity; SPE, specificity; RDW, red cell distribution width; CLIA, chemiluminescent immunoassay; PaGIA, particle–gel immunoassay; and NR, not reported.

## Data Availability

Not applicable.
